# Feasibility of a Combined Neuromodulation and Yoga Intervention for Mild Traumatic Brain Injury and Chronic Pain: Protocol for an Open-label Pilot Trial

**DOI:** 10.2196/37836

**Published:** 2022-06-15

**Authors:** Kelly A Krese, Kyla Z Donnelly, Bella Etingen, Theresa L Bender Pape, Sarmistha Chaudhuri, Alexandra L Aaronson, Rachana P Shah, Dulal K Bhaumik, Andrea Billups, Sabrina Bedo, Mary Terese Wanicek-Squeo, Sonia Bobra, Amy A Herrold

**Affiliations:** 1 Brain Innovation Center Shirley Ryan AbilityLab Chicago, IL United States; 2 Research and Development Service Edward Hines Jr Veterans Administration Hospital Hines, IL United States; 3 LoveYourBrain Foundation Norwich, VT United States; 4 Center for Innovation in Complex Chronic Healthcare & Research Service Edward Hines Jr Veterans Administration Hospital Hines, IL United States; 5 Department of Physical Medicine and Rehabilitation Feinberg School of Medicine Northwestern University Chicago, IL United States; 6 Department of Physical Medicine and Rehabilitation Edward Hines Jr Veterans Administration Hospital Hines, IL United States; 7 Mental Health Service Line Edward Hines Jr Veterans Administration Hospital Hines, IL United States; 8 Department of Psychiatry Feinberg School of Medicine Northwestern University Hines, IL United States; 9 Department of Psychiatry University of Illinois Chicago Chicago, IL United States; 10 Recreation Therapy Edward Hines Jr Veterans Administration Hospital Hines, IL United States; 11 Edward Hines Jr Veterans Administration Hospital Hines, IL United States; 12 Department of Radiology Edward Hines Jr Veterans Administration Hospital Hines, IL United States; 13 Department of Radiology Stritch School of Medicine Loyola University Chicago Maywood, IL United States; 14 Department of Psychiatry and Behavioral Sciences Feinberg School of Medicine Northwestern University Chicago, IL United States

**Keywords:** concussion, mild traumatic brain injury, chronic pain, neuromodulation, transcranial magnetic stimulation

## Abstract

**Background:**

Mild traumatic brain injury (mTBI) and chronic pain often co-occur and worsen rehabilitation outcomes. There is a need for improved multimodal nonpharmacologic treatments that could improve outcomes for both conditions. Yoga is a promising activity-based intervention for mTBI and chronic pain, and neuromodulation through transcranial magnetic stimulation is a promising noninvasive, nonpharmacological treatment for mTBI and chronic pain. Intermittent theta burst stimulation (iTBS) is a type of patterned, excitatory transcranial magnetic stimulation. iTBS can induce a window of neuroplasticity, making it ideally suited to boost the effects of treatments provided after it. Thus, iTBS may magnify the impacts of subsequently delivered interventions as compared to delivering those interventions alone and accordingly boost their impact on outcomes.

**Objective:**

The aim of this study is to (1) develop a combined iTBS+yoga intervention for mTBI and chronic pain, (2) assess the intervention’s feasibility and acceptability, and (3) gather preliminary clinical outcome data on quality of life, function, and pain that will guide future studies.

**Methods:**

This is a mixed methods, pilot, open-labeled, within-subject intervention study. We will enroll 20 US military veteran participants. The combined iTBS+yoga intervention will be provided in small group settings once a week for 6 weeks. The yoga intervention will follow the LoveYourBrain yoga protocol—specifically developed for individuals with TBI. iTBS will be administered immediately prior to the LoveYourBrain yoga session. We will collect preliminary quantitative outcome data before and after the intervention related to quality of life (TBI-quality of life), function (Mayo-Portland Adaptability Index), and pain (Brief Pain Inventory) to inform larger studies. We will collect qualitative data via semistructured interviews focused on intervention acceptability after completion of the intervention.

**Results:**

This study protocol was approved by Edward Hines Jr Veterans Administration Hospital Institutional Review Board (Hines IRB 1573116-4) and was prospectively registered on ClinicalTrials.gov (NCT04517604). This study includes a Food and Drug Administration Investigational Device Exemption (IDE: G200195). A 2-year research plan timeline was developed. As of March 2022, a total of 6 veterans have enrolled in the study. Data collection is ongoing and will be completed by November 2022. We expect the results of this study to be available by October 2024.

**Conclusions:**

We will be able to provide preliminary evidence of safety, feasibility, and acceptability of a novel combined iTBS and yoga intervention for mTBI and chronic pain—conditions with unmet treatment needs.

**Trial Registration:**

ClinicalTrials.gov NCT04517604; https://www.clinicaltrials.gov/ct2/show/NCT04517604

**International Registered Report Identifier (IRRID):**

DERR1-10.2196/37836

## Introduction

### Background

The World Health Organization estimates that the yearly incidence of mild traumatic brain injury (mTBI) is 600 per 100,000 persons and is even higher in military populations [1,2]. Although most patients are expected to recover from mTBI within 1 week to 3 months [3], a subset of patients with mTBI experience a host of poor rehabilitation outcomes, including impairments in cognition, physical health, and psychological health [4] that lead to poor quality of life (QoL) [5]. Worsening this clinical picture, 75% of individuals with TBI have comorbid chronic pain [6,7], defined as pain in the muscles, bones, ligaments, tendons, or nerves that persists for more than 6 months [8]. In addition, recent research indicates that mTBI is strongly associated with increased pain interference—a measure of the extent to which pain hinders QoL [9,10]. There are many established nonpharmacological treatment options for the sequelae of mTBI or chronic pain as individual diagnoses, including patient education, rehabilitation, and psychological interventions [11-14]. However, evidence regarding the efficacy of nonpharmacological interventions for people with co-occurring mTBI and chronic pain (mTBI+chronic pain) is lacking [15]. Further, clinical practice guidelines recommend against opioid treatment for people with mTBI+chronic pain due to an increased risk of adverse outcomes; yet, this patient cohort remains at increased risk of receiving short- and long-term opioid therapy [16]. Clinical practice guidelines for the treatment of chronic pain alone also recommend against the long-term use of opiates owing to the risk versus benefit profile [17]. The lack of sufficient evidence for any nonpharmacological intervention for mTBI+chronic pain in combination with the ongoing opioid epidemic demonstrates the strong need for effective nonpharmacological interventions for this patient population.

### Rationale

#### Yoga, Pain, and mTBI

Yoga is a mindfulness-based intervention that may be a promising alternative treatment for mTBI+chronic pain sequelae. Clinical trials of yoga interventions most commonly utilize hatha-based yoga, which is comprised of breathing exercises (*pranayama*), physical postures (*asanas*), and meditation [18]. Studies of yoga for chronic pain have found yoga to be the most effective for improving headaches and low back pain relative to pain in other areas of the body; headaches and low back pain are the most common types of chronic pain sequelae after mTBI [6,15,19-26]. The effects of yoga on various outcomes among individuals with mTBI have previously been assessed [27]. A systematic review and meta-analysis of 20 studies on yoga for mTBI revealed significant improvements in fatigue, depression, physical health, cognitive performance, and QoL [27]. One specific adaptive yoga program, LoveYourBrain yoga, is a hatha yoga program created and tested specifically for TBI and designed to be modifiable to serve people of all ability levels [28]. LoveYourBrain yoga is a manualized 6-session group-based yoga intervention that incorporates breathing exercises, physical postures, meditation, and TBI-tailored psychoeducation. LoveYourBrain yoga has been shown to be feasible in people with TBI [28-30]. Further, preliminary evidence suggests that participating in LoveYourBrain yoga leads to improvements across other outcomes, including QoL, among people with TBI of all severities [28-30]. Preliminary evidence suggests that yoga-based programs may be feasible and acceptable for individuals with mTBI+chronic pain [31]. However, the impact of yoga in general, as well as the impacts of LoveYourBrain yoga, on pain outcomes in the mTBI+chronic pain population is yet to be tested.

#### Neuromodulation and Pain

Neuromodulation through transcranial magnetic stimulation (TMS) is another promising noninvasive, nonpharmacological treatment for mTBI+chronic pain. A recent systematic review of repetitive TMS (rTMS) for chronic pain [32] and a meta-analysis of rTMS for neuropathic pain [33] demonstrated that high-frequency rTMS (>1%) applied to the motor cortex effectively reduces pain. Additionally, high-frequency rTMS applied to the left motor cortex of patients with mTBI-related headaches resulted in reductions in headache symptoms [34]. Collectively, these studies indicate that high-frequency excitatory rTMS applied to the motor cortex is beneficial for alleviating pain and can be safely and effectively applied to populations with TBI.

Intermittent theta burst stimulation (iTBS) is a type of patterned, excitatory rTMS. A practical advantage of iTBS over rTMS is that iTBS protocols are typically 3 minutes in duration instead of 30 minutes. The short duration makes this intervention ideally suited for people with chronic pain who may be unable to tolerate prolonged TMS. Research on iTBS as a treatment for pain and TBI is in its infancy. A recent trial of a single session of iTBS applied to the motor cortex of patients with chronic orofacial pain demonstrated significant, yet transient, improvement in self-reported pain [35]. These promising results may be improved with repeated provision of iTBS treatments over time. Furthermore, evidence suggests that iTBS applied to the dorsolateral prefrontal cortex may significantly decrease the frequency, duration, and severity of headaches [36], which are the most common types of chronic pain after mTBI [6,19]. Another randomized controlled trial conducted among patients with multiple sclerosis and lower spastic paraparesis examined the effects of iTBS versus high-frequency rTMS (20 Hz) on spasticity. Although researchers found that high-frequency rTMS resulted in better short-term outcomes, iTBS resulted in longer lasting improvements in outcomes [37]. These positive findings in other neurological conditions suggest that iTBS may be a promising intervention for mTBI.

At the time of publication, a single case study utilizing iTBS as a potential treatment for mTBI has been reported. The study outlined a 3-week iTBS intervention combined with rehabilitation that resulted in improvements in balance performance, motor recovery, step length, and walking speed [38]. This case study in combination with a systematic review of TMS for mTBI suggests that iTBS can be safely used among individuals with mTBI [39,40]. However, more research is essential to understanding whether this noninvasive, nonpharmacological intervention may be beneficial as a wide-scale treatment option for this population.

#### iTBS as a “Primer” for Other Interventions

What makes iTBS a unique and particularly promising intervention is that it induces a window of enhanced neuroplasticity, making it ideally suited to be paired with other interventions (eg, yoga) to magnify the effects of those interventions provided after it [41,42]. iTBS increases the excitability of the motor cortex [43] during and up to 60 minutes after the cessation of the treatment; during this time, the other intervention can be provided [43]. For example, iTBS provided prior to high-frequency (10 Hz) rTMS to the motor cortex provided greater analgesia than rTMS alone (without iTBS priming) among patients with chronic neuropathic pain [41]. In terms of pairing with behavioral interventions, iTBS was successfully combined with cognitive behavioral therapy to promote smoking cessation [42]. Collectively, this emerging evidence suggests that iTBS shows promise to prime the brain for combined interventions and may magnify the impacts that these interventions would have when used alone.

In this study, we will test the idea that for people with mTBI+chronic pain, the beneficial effects of yoga can be magnified if yoga is provided immediately after the provision of iTBS (“iTBS+yoga”). Yoga is an ideal intervention to pair with iTBS for this patient population for several reasons. A yoga program that was created specifically for TBI already exists and has been validated [28,29]. Further, comorbid mental health conditions are common in the veteran mTBI+chronic pain population owing to trauma exposure [10]. Mindfulness-based interventions such as yoga have well-established benefits for those with trauma-associated mental health sequelae [44]. Lastly, yoga is an intervention that can be easily continued independently at home after the study is complete. To the authors’ knowledge, at the time of publication, no studies have explored iTBS+yoga in any population despite burgeoning evidence in support of both of these individual interventions for a multitude of diagnoses.

#### Objectives

The objectives of this pilot study are to (1) develop a combined iTBS+yoga intervention targeting chronic pain among veterans with mTBI+chronic pain, (2) assess the intervention’s feasibility and acceptability, and (3) gather preliminary clinical outcome data on QoL, function, and pain that will guide future studies.

## Methods

### Study Design

**Figure 1:**
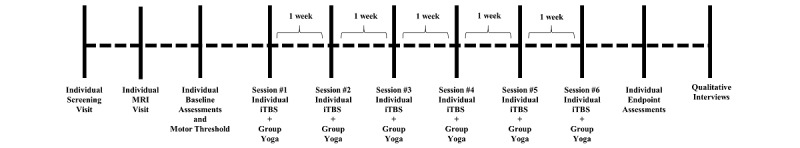
Study visit timeline. iTBS: intermittent theta burst stimulation; MRI: magnetic resonance imaging.

This study will be a single-group, exploratory, mixed methods design, wherein all participants will receive iTBS+yoga (Figure 1). The SPIRIT (Standard Protocol Items: Recommendations for Interventional Trials) recommendations were referenced when developing this protocol [45].

### Ethics Approval and Dissemination

This study protocol was approved by Edward Hines Jr Veterans Administration Hospital Institutional Review Board (Hines IRB 1573116-4) and was prospectively registered on ClinicalTrials.gov (NCT04517604). This study includes a Food and Drug Administration Investigational Device Exemption (IDE: G200195). A 2-year research plan timeline was developed (Table 1).

**Table 1:** Research plan timeline.

Task	Year 1					Year 2			
	Q1	Q2	Q3	Q4	Q1		Q2	Q3	Q4
Study team role identification	✓								
Develop intervention	✓	✓							
Participant enrollment (n)			5	5	5		5		
Study procedures			✓	✓	✓		✓		
Data analyses								✓	✓
Dissemination									✓

### Participants

We will target recruitment of 20 veterans aged 22 years or older with mTBI+chronic pain over the course of 12 months. Veteran participants must perceive themselves as able to participate in gentle physical movements and will be cleared for gentle exercise by a study team physician. mTBI will be defined according to the Veterans Affairs (VA)/Department of Defense clinical practice guidelines [46] utilizing the mTBI symptom attribution and classification algorithm (SACA) [47]. Chronic pain will be operationally defined as pain in the muscles, bones, ligaments, tendons, or nerves that persists for >6 months and is of moderate-to-severe intensity as indicated by a score of >5 on specific items on the Brief Pain Inventory [8,48]. For safety, we will exclude persons with contraindications to TMS or magnetic resonance imaging (MRI). We will not exclude participants with major depressive disorder and other psychological disorders; these diagnoses will be documented as potential covariates. We will not exclude participants based on gender, ethnicity, or race. All participants will provide written informed consent in accordance with the World Medical Association Declaration of Helsinki. Each participant is eligible to receive compensation for participation. The inclusion and exclusion criteria are listed below (Textbox 1).

**Inclusion and exclusion criteria for the participants.**

**Inclusion criteria**

- 22+ years of age
- Can read and speak English
- Perceive themselves as able to participate in gentle physical movements and cleared by study physician to do so.
- Mild traumatic brain injury (mTBI) criteria: Symptom Attribution and Classification criteria for mTBI (without requirement of clinical neuropsychological impairment)
- Chronic pain: pain (in muscles, bones, ligaments, tendons, or nerves) that persists for >6 months and is of moderate-to-severe intensity with a score of >5 on specific items on the Brief Pain Inventory
- Fully vaccinated against COVID-19 prior to study participation

**Exclusion criteria**

- Contraindications to intermittent theta burst stimulation/transcranial magnetic stimulation (eg, epilepsy, history of anoxic brain injury, or heart disease)
- Contraindications to magnetic resonance imaging (eg, claustrophobia, ferromagnetic metal implants)
- Pain believed to be associated with cardiac or ischemic conditions
- History of moderate-to-severe TBI
- Active seizure disorder or if they are taking psychostimulants (eg, amphetamines), anticholinergics, or other medications that may increase their risk of having seizures
- History of or current psychosis not due to an external cause (eg, due to illicit drug use)
- Are pregnant or nursing
- Within 12 weeks of a major surgery/operation
- Have questionably valid test profiles

### Recruitment

We will recruit veterans from a large VA medical center located in the midwestern United States, which houses a TBI/Polytrauma program that admits approximately 250 veterans per year. TBI/Polytrauma program personnel will inform the program members about the study through routine staff meetings. TBI/Polytrauma program personnel will add research team members as co-signers in the electronic medical record (EMR) of TBI/Polytrauma program patients recommended for screening for this study. We will then perform an EMR review for study eligibility criteria and send eligible veterans an informational letter about the study. We will make follow-up phone calls to perform initial eligibility screens. We will also mail informational letters to veterans who have completed our past studies and permitted us to contact them about future studies through a TBI Data Repository created for recruitment purposes (coprincipal investigators: AAH and TLBP, IRB#14-003).

### Data Collection and Measures

#### MRI Data Acquisition, Neuronavigation, and Motor Thresholding

All participants will undergo a brain MRI. A high-resolution, 3D, T1-weighted, multigradient-echo sagittal anatomical scan (voxel size=0.8 mm isotropic resolution) will be collected in order to allow for iTBS treatment site neuronavigation for each participant. The images will be reviewed by a neuroradiologist. In order to ensure the MRI procedure will be safe, the participant will be asked to fill out a standardized MRI safety screening form before starting the study. Examples of potentially exclusionary issues include (1) metal fragments in the eyes or face, (2) implantation of any electronic devices such as (but not limited to) cardiac pacemakers, cardiac defibrillators, cochlea implants, or nerve stimulators, (3) surgery on the blood vessels of the brain or the valves of the heart, (4) claustrophobia, and (5) body piercing or tattoos.

Each participant’s T1 MRI will be loaded into a Localite TMS Neural Navigator system to identify his or her motor threshold. A MagVenture C-B60 coil will be used to deliver single-pulse TMS to the nondominant motor cortex to identify the abductor pollicis brevis muscle brain coordinates. Stimulation intensity that will be used in iTBS will be determined by collecting each participant’s motor threshold using the finger representations of the motor cortex. The consensus in the literature is that iTBS can be safely provided at 80% of active motor threshold (AMT) [49-53]. Since there is more within- and between-subject variability with AMT (eg, different gripping strengths) relative to resting motor threshold (RMT), scientifically, the RMT is preferred. There is also recent evidence that motor threshold estimates using RMT and AMT are equivalent [54]. This means that treatment intensity based on these 2 motor threshold estimation procedures would be equivalent. We will use RMT to estimate motor threshold and compute treatment intensity. RMT will be defined as the lowest stimulus intensity necessary to produce motor-evoked potentials ≥50 µV in 5/10 trials. Thus, the standard iTBS parameters will be used in this trial to maximize safety. iTBS will be provided at 80% of RMT.

#### Quantitative Data Collection

Information that will be collected via pre-enrollment telephone screening to assess for eligibility includes (1) probable mTBI, using the Ohio State University TBI Identification Method [55], (2) MRI safety, using a standardized hospital-wide MRI safety form, (3) self-reported age (to be verified in the EMR) and ability to read and speak English, and (4) a list of prescribed and over-the-counter medications and the length of time the participant has been on each medication (Table 2).

**Table 2:** Quantitative study assessments.

Assessment name			Assessment purpose
**Eligibility phone screen**			
	Ohio State University TBI^a^ Identification Method	Identification of likelihood of mTBI event	
	Hines Veterans Affairs magnetic resonance imaging safety form and transcranial magnetic stimulation safety form	Magnetic resonance imaging and transcranial magnetic stimulation safety compatibility	
**Initial screening**			
	Structured Diagnostic Interview [47] with the Neurobehavioral Symptom Inventory [56]	mTBI eligibility	
	California Verbal Learning Test-II [57]	Memory and performance effort validity	
	Minnesota Multiphasic Personality Inventory-2-Restructured Form [58]	Symptom reporting validity	
	Demographics	Sample characterization	
**Baseline and endpoint**			
	TBI-quality of life (TBI-common data elements)	Preliminary quality of life data	
	Mayo-Portland Adaptability Index (TBI-common data elements)	Preliminary function and participation data	
	Brief Pain Inventory [8,48]	Preliminary pain data and eligibility	
	Therapy Activity Data Collection	Preliminary Therapeutic Activity Data	
	Session completion rates	Feasibility (endpoint only)	
	Structured qualitative interviews	Acceptability (endpoint only)	
	Satisfaction ratings	Acceptability (endpoint only)	
	Home and community yoga and meditation diaries	Covariate (each weekly session)	

^a^TBI: traumatic brain injury.

Prior to study enrollment, we will conduct in-person screening with each potential participant, during which veterans will complete a finite set of assessments from the mTBI SACA [47]. The Structured Diagnostic Interview, included in the SACA, will be used to establish the mTBI history, including duration of loss of consciousness, alteration of consciousness, and posttraumatic amnesia [47]. The interview ends with the Neurobehavioral Symptom Inventory [56]. The California Verbal Learning Test-II (CVLT-II) [57] will be used to assess verbal memory, a cognitive domain affected by mTBI. To determine the validity of the test profiles, a cutoff score of 15 on the CVLT-II forced choice component will serve as a measure of effort performance [59]. To determine the validity of symptom reporting, the Minnesota Multiphasic Personality Inventory-2-Restructured Form [58] will be used to identify abnormal symptom reporting via this criteria: F, T score≥107; F(p), T score≥85; True Response Inconsistency, T score≥80; and Variable Response Inconsistency, T score≥80 [47].

Baseline and endpoint assessments, which will be collected at the participant’s third and tenth visits include TBI common data elements (ie, TBI-related QoL, Mayo-Portland Adaptability Index) and the Brief Pain Inventory. The TBI-QoL is a self-report instrument developed by TBI Model System centers and VA Brain Injury Centers that assesses domains of TBI-specific functioning and health-related QoL [60]. The Mayo-Portland Adaptability Index is an outcome of abilities, adjustment, and participation that is validated for use in veterans with TBI [61]. The Brief Pain Inventory is a self-reported measure assessing the extent to which pain interferes with domains of functioning such as mood, work, sleep, and enjoyment of life [48].

At the endpoint visit, participants will also complete a 2-question satisfaction rating scale [28], which serves as a quantitative assessment of intervention acceptability. The questions include (1) “would you recommend this program to a friend?” and (2) “on a scale of 1 (poor) to10 (excellent), how would you rate the quality of this program?”

Feasibility will be defined by enrollment and the number of sessions completed by each participant. Participants will be instructed to complete weekly diaries throughout the intervention phase of the study detailing pain medication usage, other pain management strategies utilized, and the time they spent at home or in the community engaging in meditation or yoga practices. These factors will be used as a potential covariate in analyses.

#### Qualitative Data Collection

The acceptability of the intervention will be qualitatively assessed via semistructured interviews with a subsample of veterans who participate in the study (n=10). Interviews will elicit feedback about veteran experiences with intervention participation, barriers and facilitators to participation, perceptions of the intervention, how participation impacted key pain-related outcomes and QoL, and suggestions for improvement.

#### Safety and Adverse Event Monitoring

Participants will participate in safety monitoring using the Data Safety Monitoring Scale before and after each iTBS session. This scale assesses vital signs (temperature, blood pressure, heart rate, oxygen saturation levels), fatigue, tinnitus (ringing in the ears), hours of sleep, dizziness, nausea, vomiting, confusion, seizure, syncope (fainting), headache, neck pain, skin integrity of the scalp, and substance use and directs how staff should respond if any of the values deviate significantly from the individual’s baseline values.

An adverse event is any undesirable experience associated with iTBS measured as a deleterious change from baseline on the Data Safety Monitoring Scale. A serious adverse event is when the changes are disabling, life threatening, require hospitalization, or require intervention to prevent impairment. We will measure deleterious changes in (1) neurologic status, including cognitive symptoms, (2) somatic and vestibular symptoms, (3) and depression. Adverse events will be tracked using an adverse event log.

All iTBS sessions will be videotaped to allow for review in case of an adverse event. Acknowledgement for picture and video will be completed as part of Health Insurance Portability and Accountability Act (HIPAA) authorization documentation.

All serious, unanticipated adverse events that are related to this research study will be reported to the institutional review board within 5 days. If any unanticipated problem occurs, such as deviation from this protocol that involves risks or has the potential to recur, this information will be reported by the investigator to the institutional review board as well within 2 business days but no longer than 5 business days of the investigator or staff becoming aware of the event.

Any concern for seizure or seizure-like activity will result in stopping treatment and, if indicated, the participant will be withdrawn from the study. The on-site rapid response team will be paged to provide emergent medical care to the participant, including administration of seizure-abating medications and airway protection, if necessary. If emergent medical care is required, we will transport the patient to the hospital emergency room for further monitoring. All research team members have been educated on how to safely lower patient to the floor prior to the rapid response team arriving. If the participant ultimately does not require emergent care, they will be advised to follow up with their established health care provider. If concerning new conditions emerge or existing conditions worsen, we will withdraw the participant from the proposed study.

### Experimental Interventions

#### iTBS Intervention

We will use a T1 MRI to localize the stimulation site, which will be each participant’s dominant trunk representation area of the motor cortex. The 3-minute iTBS protocol will be delivered utilizing the MagVenture Mag-Pro X100 with the MagOption stimulator that includes active and placebo coils (C-B60 Butterfly coils) and equivalent active only Cool-B65 Butterfly treatment coil. Only the active setting will be used. iTBS parameters include 3 pulses of stimulation given at 50 Hz, repeated every 200 ms at 80% of the RMT. The interpulse interval is 20 ms. A 2-second train of TBS is repeated every 10 seconds for a total of 190 seconds, which equates to a total of 600 pulses [62].

#### LoveYourBrain Yoga

**Figure 2:**
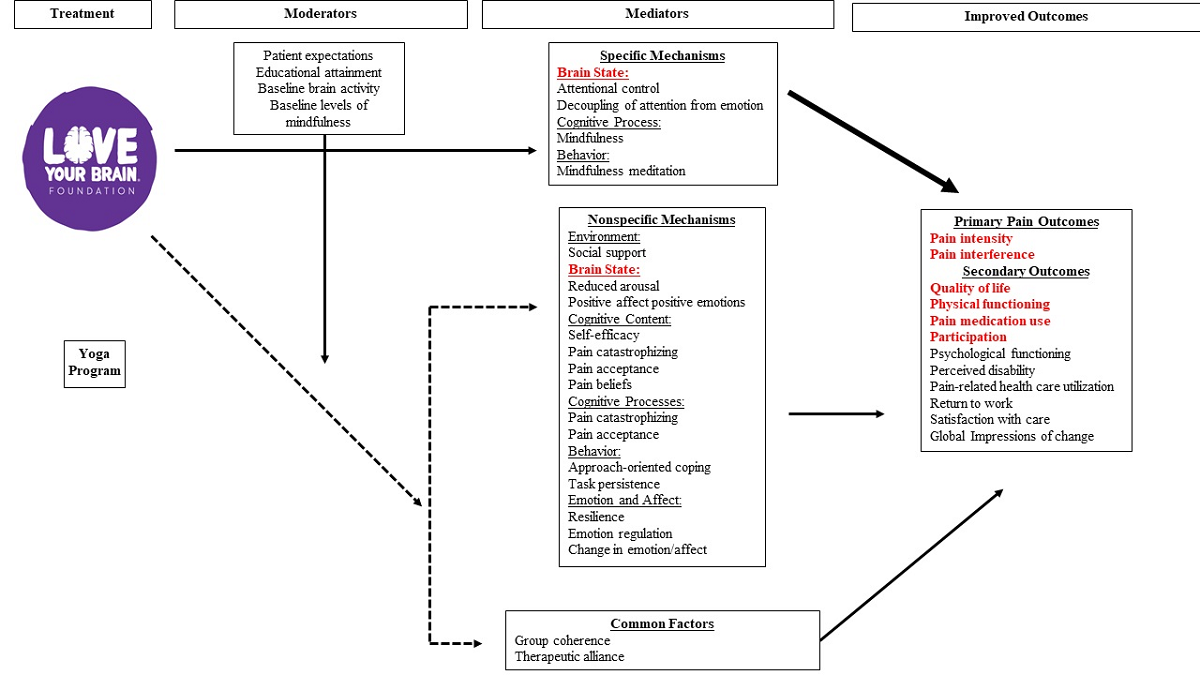
Adapted conceptual model of the mechanisms of mindfulness-based interventions for chronic pain management [63]. The mediators in bold red font represent those which we are targeting with intermittent theta burst stimulation paired with the LoveYour Brain yoga program. The outcomes in bold red font are those which we will formally assess at baseline and endpoint.

The LoveYourBrain yoga curriculum includes 6 weekly 90-minute sessions comprised of 10 minutes of breathing exercises, 45 minutes of gentle yoga, 15 minutes of guided meditation, and 20 minutes of facilitated discussion with psychoeducation. The program is detailed in a manual to ensure standardization of program content across time and instructors [28]. We adapted the existing psychoeducation component to include pain-related topics. We engaged key stakeholders to assist in the psychoeducation tailoring process, including the LoveYourBrain faculty and developers, TBI/Polytrauma and pain clinic teams at the study site, veterans, and the individuals from the pain management center at a nearby large rehabilitation hospital. Day et al’s [63] conceptual model of the mechanisms for improvements after mindfulness-based interventions for chronic pain management informed our tailoring of the existing psychoeducation program to optimally engage those with pain (Figure 2). The LoveYourBrain yoga psychoeducation is already focused on resilience, which Day et al’s model [63] identifies as a mechanism of improvement in pain after an intervention. Based on Day et al’s [63] conceptual model, we also integrated pain beliefs, acceptance, and self-efficacy into the existing psychoeducation program.

Each LoveYourBrain yoga session will be led by 2-3 instructors. Each instructor will complete the 20-hour LoveYourBrain yoga Teacher Training certification course to learn how to deliver the LoveYourBrain yoga and adapt yoga, breathing exercises, meditation, and group discussion for TBI. The asana portion of the program will be led by certified yoga teachers. To support fidelity of intervention delivery, instructors will be required to submit recordings of mock teaching the LoveYourBrain yoga curriculum to LoveYourBrain faculty and receive detailed feedback. Instructors will also utilize LoveYourBrain’s session flow sheets to ensure intervention consistency and fidelity.

### Study Procedures

A partial HIPAA waiver and waiver of informed consent for screening purposes will provide regulatory approval to screen candidates to determine study eligibility prior to obtaining informed consent. After the eligibility phone screening, we will cross-reference current medications with study eligibility criteria and the EMR to identify any antiepileptics and medications known to lower seizure threshold. If findings indicate possible contraindications to rTMS or MRI related to a metal implant, we will obtain the model and manufacturer of the implant to determine whether it is safe to expose the implant to a strong magnet. We will follow manufacturer recommendations regarding safety. If we do not identify any exclusionary factors in the eligibility phone screening, we will invite the participant to complete an in-person screening.

Several outcomes will be completed at the initial in-person screening (Table 2). After the screening, a study physician will evaluate the veteran’s ability to engage in gentle exercise and their pain management strategy, including over-the-counter analgesics that they might take during the intervention. Participants will still be able to receive other potential treatments or medications to assist with the management of chronic pain. These pain management strategies must remain stable during study participation. Upon the initial evaluation and at each study visit, medication dosages and frequencies will be documented.

Intervention sessions will occur once a week for 6 weeks, with approximately 2-4 individuals participating simultaneously as a cohort. Prior to the interventions, participants will provide a urine sample for the detection of alcohol and certain drugs that lower seizure threshold. This includes pain medications such as morphine and Vicodin; benzodiazepines such as Valium, Librium, Xanax, and Ativan that are usually used to treat anxiety or alcohol withdrawal; and substances such as cocaine, marijuana, heroin, amphetamine or speed, and barbiturates. These screening results are only used to determine if participants are eligible to continue in this research study and will not be entered into the participant’s medical record or reported to legal authorities.

Each intervention will start with individual iTBS for each of the approximately 2-4 participants per cohort. The order of receipt of iTBS will be counterbalanced so that each individual receives iTBS first, second, third, or fourth as close to the same number of times as possible. While not receiving iTBS, the participants will be instructed to rest quietly in another research space or waiting area. Each iTBS session will take about 15 minutes with setup and takedown.

Ear plugs will be placed in participants’ ears for protection since the magnetic stimulator makes a loud clicking noise. Because of the known potential for pain associated with iTBS, we will recommend that participants bring an over-the-counter pain reliever of their choice to take prior to each iTBS session. We will ask participants when they took the medication and the amount.

Within 60 minutes of the first individual’s receipt of iTBS, a 90-minute teacher-guided 2-4 person group LoveYourBrain yoga session will be initiated. If a participant misses a session, they will be contacted within 24 hours to determine reasons for missed sessions, which will also be tracked in an activity log. The participant will then be encouraged to make up for their missed session, which will be offered as an optional group makeup session at the end of the 6-week program. If participants miss less than 50% of the sessions (3/6), they will have the opportunity to make up the missed sessions. If the participants miss 3 or more sessions, they will have the opportunity to join the next cohort.

### Analytic Plan

#### Quantitative Analysis

For each outcome, we will perform a paired, 2-sided *t* test between baseline and endpoint scores (α=.05). To measure the pairwise association between 2 outcomes, we will compute Pearson correlations and test for significance by using the Fisher Z-transformation. We will also examine the relationship between number of completed sessions and change score (endpoint – baseline) of each outcome with a linear regression model. We will extract information from this pilot study and compute effect sizes used for sample size determination in future studies based on the change score for each outcome and compute the sample size for 80% power with a 5% type 1 error rate.

#### Qualitative Analysis

Semistructured interviews will be audio recorded, transcribed verbatim, and analyzed by 2 qualitative experts by using thematic coding and constant comparison techniques. Our qualitative analysis will be supported by a codebook, the initial content of which will be guided by the elements of our conceptual model. Each interview transcript will be independently coded by 2 members of the research team; these individuals will then meet to discuss codes and resolve discrepancies as needed.

## Results

As of March 2022, we have enrolled 6 individuals. We will collect data until November 2022. We expect the results of the study to be available by October 2024.

## Discussion

### Hypotheses

Our central hypothesis is that our combined iTBS+yoga intervention will be feasible and acceptable for veterans with mTBI+chronic pain. This hypothesis is based on existing literature and our preliminary data demonstrating that iTBS [38] and the LoveYourBrain yoga program [28], provided separately, are each feasible and acceptable among people with TBI. Further, we hypothesize that the relationship between iTBS+yoga and all outcomes will be positive, for example, more sessions will provide better change scores.

It is expected that veterans with mTBI+chronic pain will be able to attend the weekly sessions for 6 weeks. The quantitative satisfaction ratings will improve by the completion of the treatment. The semistructured interviews and feedback will allow for modifications to be made as warranted. Although we expect that the 3 outcome domains of QoL, function, and pain will improve, even if a single domain improves, we consider this positive. By exploring the associations between the above outcomes, we will better be able to interpret which outcome domain may be driving improvements.

Pending the results of this pilot trial, larger scale randomized controlled trials may be warranted to determine the efficacy of iTBS+yoga on improving QoL, function, and pain among veterans with mTBI+chronic pain.

### Strengths of This Study

The strengths of this study are as follows. First, this study utilizes an established program already vetted for feasibility and acceptability for the population with mTBI. Second, both iTBS and yoga are readily available within the VA health care systems throughout the United States.

### Limitations of This Study

The limitations of this study could be as follows:

1. The small sample size reduces the study power, which can limit the ability to interpret the significance of any differences found in outcomes but is reflective of the pilot nature of the study and the funding mechanism.
2. The open-label nature of this study could bias results but is also an important first step for developing this novel intervention.
3. Variability in the baseline characteristics of the patient population that are not being studied in detail in this project may negatively impact some measures of QoL and pain. Thus, we may explore treatment responsiveness by baseline characteristics.
4. Pain is a truly subjective measure; therefore, we will explore calibrating self-report pain outcomes on the basis of function and QoL outcomes.

## Appendix

Multimedia Appendix 1 Peer-review report by the Rehabilitation Research and Development Small Projects in Rehabilitation Research (SPiRE) Program - Rehabilitation Research and Development Parent IRG, Office of Research & Development (RRDS) - United States Department of Veterans Affairs.

## Abbreviations

AMT: active motor threshold
CVLT-II: California Verbal Learning Test-II
EMR: electronic medical record
HIPAA: Health Insurance Portability and Accountability Act
iTBS: intermittent theta burst stimulation
iTBS+yoga: intermittent theta burst stimulation paired with the LoveYourBrain yoga program
MRI: magnetic resonance imaging
mTBI: mild traumatic brain injury
mTBI+chronic pain: co-occurring mild traumatic brain injury and chronic pain
QoL: quality of life
RMT: resting motor threshold
SACA: symptom attribution and classification algorithm
SPIRIT: Standard Protocol Items: Recommendations for Interventional Trials
TMS: transcranial magnetic stimulation
VA: Veterans Affairs
