# Supplementary material for: Feasibility of a Combined Neuromodulation and Yoga Intervention for Mild Traumatic Brain Injury and Chronic Pain: Protocol for an Open-label Pilot Trial
Source: JMIR Res Protoc. 2022 Jun 15;11(6):e37836. doi: 10.2196/37836 (PMC9244651; doi:10.2196/37836)
Supplement: Multimedia Appendix 1 [file resprot_v11i6e37836_app1.pdf]

SUMMARY STATEMENT

PROGRAM CONTACT: (Privileged Communication) *Release Date:* 05/13/2020

Revised Date:

Application Number: 1 I21 RX003611-01

Principal Investigator

HERROLD, AMY

Applicant Organization: EDWARD HINES JR VA HOSPITAL

Review Group: RRDS
Rehabilitation Research and Development SPiRE Program

Meeting Date: 04/23/2020 *RFA/PA:* RX20-009
Council: AUG 2020
Requested Start: 10/01/2020

Project Title: Feasibility of a Combined Neuromodulation and Yoga Intervention for Veterans with Mild Traumatic Brain Injury and Chronic Pain
SRG Action: Impact Score:233

Human Subjects: 30-Human subjects involved - Certified, no SRG concerns
Animal Subjects: 10-No live vertebrate animals involved for competing appl.
Gender: 1A-Both genders, scientifically acceptable
Minority: 1A-Minorities and non-minorities, scientifically acceptable
Age: 1A-Children, Adults, Older Adults, scientifically acceptable
Clinical Research - not NIH-defined Phase III Trial

Project Year	Direct Costs Requested
1	100,000
2	100,000
TOTAL	200,000

CONDITIONS:

- In order to minimize confounds associated with inconsistent timeline of interventions, clarify how much time will lapse between iTBS and start of yoga intervention for each participant.
- Indicate the yoga instructor's qualifications/training to perform the yoga sessions.
- Describe how administration of an analgesic before and/or after intervention will be controlled and accounted for in any outcomes measured. Provide the protocol for analgesic administration.

ADMINISTRATIVE NOTE:
The presence of a condition(s) is not an indication that the application has been selected for funding. If a notice of intent to award is issued, then the condition(s) will need to be addressed as part of Just-in-Time (JIT).

DESCRIPTION (provided by applicant):
Over 340,000 people have incurred a mild traumatic brain injury (mTBI) as a result of the military conflicts in Iraq and Afghanistan. mTBI leads to a host of poor rehabilitation outcomes including impairments in cognition, physical health, and psychological health. These impairments among people with TBI lead to poor quality of life (QOL). Worsening this clinical picture, the prevalence of chronic pain is estimated to be 51.5% among civilians with TBI and 43.1-70% among Veterans with TBI. Opioids are used for treating chronic pain including among people with TBI. Thus, given the ongoing opioid epidemic in the United States, it is very timely to develop alter- native, non-pharmacologic treatments for chronic pain among Veterans with mTBI. Yoga is a promising activity-based intervention for TBI and chronic pain. Yoga is an activity generally comprised of breathing exercises, gentle stretching, and meditation. Neuromodulation through transcranial magnetic stimulation (TMS) is a promising non-invasive, non-pharmacological treatment for TBI and chronic pain. Intermittent theta burst stimulation (iTBS) is a type of patterned, excitatory TMS. iTBS can induce a window of neuroplasticity, making it ideally suited to boost the effects of treatments provided after it. Thus, iTBS shows promise to prime the brain for combined interventions and may magnify the impacts that these interventions would have when used alone, in order to boost outcomes. The purpose of this SPiRE project is to develop a novel, combined neuromodulation and yoga neurorehabilitation intervention for Veterans with mTBI and chronic pain, and to examine the intervention's feasibility and acceptability. Using an existing, evidence-based, yoga program created specifically for people with TBI (LoveYourBrain Yoga), we will first develop a neurorehabilitation intervention that combines iTBS with yoga (iTBS+yoga), and then collect pilot data about its feasibility and acceptability. Aim 1 will develop a novel, combined iTBS+yoga neurorehabilitation intervention for Veterans with mTBI and chronic pain. Aim 2 will examine the feasibility and acceptability of the iTBS+yoga intervention for Veterans with mTBI and chronic pain. Aim 3 will gather preliminary data to provide the foundation for sample size and power considerations for a future clinical trial to examine the effectiveness of iTBS+yoga on Veterans' quality of life, function and pain outcomes. The combined intervention will be provided in small group settings once a week for 6 weeks. iTBS will be administered immediately prior to the LoveYourBrain Yoga session. Emphasizing National Institute of Neurological Disorders and Stroke (NINDS) Traumatic Brain Injury Common Data Elements (TBI CDEs), we will collect preliminary outcome data related to quality of life, function and pain to inform a future Merit application, should the intervention prove feasible. This SPiRE project will directly benefit Veterans and VA Services by developing a new, non-pharmacological neurorehabilitation treatment for Veterans with mTBI and chronic pain in need of non-opioid treatment options. TMS is now offered at 30 VA hospitals nationwide for treatment-resistant depression, and yoga is among the complementary and integrative health programs being rolled out as a part of VAs nation-wide Whole Health implementation efforts, with classes offered through VA service lines such as recreational therapy. Therefore, should iTBS+yoga ultimately prove to be efficacious and effective, VA facilities will be well-poised to offer this treatment. A novel, activity-based, non-pharmacological

treatment for Veterans with mTBI and chronic pain is of great need given the high prevalence of chronic pain, increased risk of opioid therapy, and increased risk of developing opioid use disorders.

PUBLIC HEALTH RELEVANCE:
The objectives of this VA SPiRE application are to develop a combined neuromodulation and yoga (iTBS+yoga) intervention for Veterans with mild traumatic brain injury (mTBI) and chronic pain, assess the intervention's feasibility and acceptability, and to gather preliminary clinical outcome data on quality of life, function and pain that will guide future studies. This SPiRE project will directly benefit Veterans and VA Services by developing a new, non-pharmacological neurorehabilitation treatment for Veterans with mTBI and chronic pain in need of non-opioid treatment options. Neuromodulation is now offered at 30 VA hospitals and yoga is among the complementary and integrative health programs being rolled out as a part of VAs Whole Health implementation efforts. Thus, should iTBS+yoga ultimately prove to be efficacious, VA facilities will be well-poised to offer this treatment. A novel, activity-based, non-pharmacological treatment for Veterans with mTBI and chronic pain is of great need given the high prevalence of chronic pain.

CRITIQUE 1

Significance:
- Highly significant in context of improving non-opioid pain management options for Veterans.
- With extant research supporting use of yoga for management of pain, concept of creating an intervention that magnifies impact of yoga intervention is novel and interesting.
- A successful intervention of this type would likely be very significant in terms of increasing VA treatment options for common medical concerns experienced by the Veteran population; could have cost-effectiveness outcomes for VA healthcare systems as a whole.
- If the combined model seems feasible, acceptable, and beneficial, it can be tested later with other well-established interventions for different (and potentially hard-to-treat) medical issues.

Approach:
- Aim 1: Develop a novel, combined intervention (iTBS + Yoga) for Vets with TBI and Chronic Pain.
  - Aim 1 seems well-developed; the iTBS and the LoveYourBrain Yoga protocols are well-established on their own. The researchers are proposing to combine them. One question is – will all participants in each group be given the iTBS at the same time? In the proposal as written it is unclear if the timeline for application of iTBS prior to starting yoga will vary between participants. In order to minimize confounds associated with inconsistent timeline of interventions, please clarify how much time will lapse between iTBS and start of yoga intervention for each participant.
- Aim 2: Examine feasibility and acceptability of the novel intervention.
  - Aim 2 seems like an important step for future research. Researchers seem to have an *a priori* hypotheses that the intervention will be feasible and acceptable to the population based on extant research utilizing iTBS and LoveYourBrain yoga with populations of patients with TBI.
  - It will be important to consider potential barriers (e.g., psychosocial, physiological, psychological) to sustaining attendance at a yoga class for 6 weeks. There exists some research surrounding such barriers.
  - Per the protocol, "acceptability will be assessed using…semi-structured interviews with a sub-sample of 10 Veterans." Why not do the interview with all Veterans who completed the study given that the study proposes recruiting only 20 Veterans. If there is a reason for assessed acceptability/feasibility via only half of the study population, please provide this rationale.

    - It is very impressive that the research team has already conducted preliminary acceptability outreach and has incorporated Veteran feedback where appropriate.
- Aim 3: Gather preliminary data about QOL and pain outcomes to inform future research.
    - Patients with TBI may struggle with sustaining attention. Individuals with chronic pain are also prone to concentration difficulties. Thus, the battery off screeners and assessments may require an ability to sustain attention for a period that may exceed the capacity of the typical TBI/chronic pain patient. It is recommended that the battery of screeners proposed to assess outcomes be pared down as much as possible to reduce participant burden. In addition, may be important to consider burden & feasibility of keeping "weekly diaries" in this population.

Innovation:
- The proposed study appears to combine two increasingly available interventions (TMS offered at 30 VA hospitals; yoga with Whole Health at VA's) in a very creative way. The proposal seems quite innovative and, if results suggest the proposed combined intervention could be beneficial for Vets with TBI + Chronic Pain, the innovation will likely have a significant and far-reaching impact.
- Per the proposal, the impact of LoveYourBrain yoga nor the impact of iTBS has ever been tested on pain outcomes in a population of patients with TBI.

Investigator Qualifications:
- Dr. Herrold and her team members appear highly qualified to engage in the proposed study.
- Need to clarify who will be leading the LoveYourBrain yoga: does this person have training/qualifications in yoga instruction? Medical/anatomy training? It may be helpful to provide more information about the actual postures involved in this yoga protocol as well to ensure that it is a protocol that can be implemented by an adequately trained instructor.

Environment:
- All aspects of proposed environment seem adequate for supporting this research; the investigators have access to all needed equipment, software, consultants, etc.
- Collaboration between Hines VA and Northwestern University seems ideal and likely to provide all necessary resources.

Design/Scope:
- Recruitment: Procedures seem acceptable and in accordance with VA guidelines.
- Screening Visit: Likely too long/too much burden associated with number of screeners/assessments. May need to break this session into two to reduce burden and assist patients in sustaining attention and effort.
- MRI visit: No concerns.
- iTBS & Yoga Intervention:
    - Need to ensure that time lapse between iTBS application and start of yoga session is equal for all patients or is statistically controlled for in outcomes.
    - Need to clarify who is leading the yoga session and what the training of this individual will be. Whereas many individuals may be able to lead a meditation and psychoeducation session, there are aspects of leading a 45-min gentle yoga session that will require an instructor to have adequate training if it is to be done safely in this population.
    - Also, will it be the same set of participants in each intervention group each time? Or will the participants be allowed to switch group days/times each week? If group attendees are not the same in each group each week there will need to be a discussion as to why this is allowed (e.g., making up a missed session) and a plan to account for social influence differences (e.g., outcomes during a yoga session may be very different in different groups of people).

   - Protocol suggests that participants will be “given acetaminophen before each iTBS session to reduce any potential discomfort.” Will acetaminophen be given at every iTBS session for every participant? What about those for whom a dose of acetaminophen is contraindicated? Will there be an alternative NSAID option? What happens when someone does not take the NSAID offered? The protocol states that patients may also be allowed to take a 2nd dose of the medication if needed. In any population – and in a chronic pain population especially -the application of a pain medication before engaging in the yoga intervention is likely to play a role in the person’s ability to engage in the yoga practice. The application of this medication after the yoga practice is also likely to influence someone’s perception/satisfaction associated with said intervention. The application of an NSAID before and/or after intervention should be controlled and accounted for in any outcomes measured.
- Qualitative analysis: Procedures are appropriate; however, need rationale for using only half of sample for this piece.
- Quantitative analysis:
   - If missing data is noted and cannot be inputted, what will happen with those missing data points? Please elaborate on this statistical plan.
   - In addition, please clarify plan for accounting for inflated Type 1 error in light of the proposed plan to perform a series of t-tests.

Human Subjects:
- The study proposes to enroll 20 Veterans >22 years of age (assuming that individuals under that age will not yet qualify as a Veteran?) who have both mild TBI and chronic pain.
- Would strongly recommend having some form of medical clearance required for each participant.
- Inclusion & Exclusion criteria listed seem generally appropriate.
- Additional criteria to consider include pre-existing experience with yoga/meditation and current use of opioid (or like) pain medications. If individuals with previous yoga experience will not be excluded, please consider controlling for impact of said previous experience and pre-existing positive views on the potential benefit of yoga may have. In addition, individuals who are vs. are not taking pain medications at the time of this study are likely to have very different experiences. This should be accounted for.

Inclusion of Women, Minorities and Children:
- Participants will be age 22 or older.
- Women and minorities will not be excluded, though pregnant or nursing women will be excluded.

Critique of Vertebrate Animals Section: Not applicable.

	Yes	No
Research with vertebrate animals?		X

Biohazards and Radioisotopes: No concerns.

Budget: Please clarify where the $190 that participants are “eligible to receive” will come from, when/how this will be awarded, and if participants are aware of this potential at onset of study.

Data Management and Access Plan (for data sharing, unscored)**:**
- Data de-identification and confidentiality procedures seem appropriate.
- Data sharing between VA researchers and Northwestern University seems appropriately planned.

Overall Strengths:
- Novel intervention idea with potential for broad significant impact in a population that needs alternative interventions for these common medical comorbidities.
- Creative collaboration between many medical/research personnel at two sites.
- Important steps to take to move forward with research for alternative pain management interventions.
- Assists in legitimizing the utilization of yoga-based interventions in medical populations.

Overall Weaknesses: Some concerns surrounding procedure and protocol that need to be controlled for/accounted for before data can be accurately interpreted.

CRITIQUE 2

Significance: This proposal is appropriate for the SPiRE funding mechanism due to the potential clinical utility of the intervention. Chronic pain is highly prevalent among Veterans and there is a critical need for effective, nonpharmacological interventions to improve functional outcomes.

Approach: The approach is to combine two interventions to reduce chronic pain and improve quality of life in Veterans with MTBI, so the proposal has clear relevance to clinical care. However, the small sample size, lack of any control subjects, the relatively high burden on participants (all of whom have chronic pain) and the combination of three interventions (iTBS, yoga and a cognitive/psychosocial intervention) will make it difficult to determine which aspects of the treatment are effective. For this funding mechanism, it might be more effective to simplify the approach and increase the number of subjects rather than attempting to understand the effects of multiple interventions with such a small group of subjects. Given the required participation (initial assessment, MRI, 6 weekly 2 hour sessions and keeping a diary, plus multiple post-assessments) there is a risk of drop outs or partial participation given the nature of chronic pain. Enrolling a greater number of subjects will help account for the possibility of treatment drop-out. The iTBS appears to have a sham setting which would be very helpful if you used a randomized design (with and without iTBS) and all subjects could complete the yoga and psychosocial intervention, making the results very useful in determining if the iTBS has added benefit to the yoga. This is important because yoga is a relatively low-cost intervention which can be provided across the VA, while iTBS requires significant investments in equipment, neuroimaging resources and time of the subject.

Innovation: This study is attempting to validate several non-pharmacological interventions by assessing pre and post-measures of clinical and functional status in Veterans with chronic pain and MTBI. By studying a novel way (iTBS) to potentiate the effects of a behavioral intervention, this study is innovative. Improving our understanding of the clinical utility of iTBS as an adjunctive to behavioral interventions is exciting and potentially very valuable. This is a non-invasive treatment which combined with yoga could have a high impact on treatment options for chronic pain.

Investigator Qualifications: This investigator has excellent experience and appears very well qualified for this study. She has a number of good collaborators on her study and she is well positioned within the institution to obtain necessary support and expertise to accomplish this study.

Environment: Not applicable.

Design/Scope: The design of the study is a limiting factor as written. The study design will not allow the investigator to determine if any effects are due to the iTBS, the physical intervention or the

psychosocial intervention. Improving the study design to examine the yoga using a small sample or increasing the sample size and using a randomized control of a sham iTBS intervention with the yoga+psychosocial intervention would greatly improve the strength of the study and determine if the iTBS has added value in improving quality of life, function and reducing chronic pain in Veterans.

Human Subjects: The safeguards and recruiting of participants is well described and subjects will be compensated, but the time commitment and number of appointments involved in this study will be a limiting factor for recruitment efforts. Even if appointments are offered at various times during the day, this will also add a potential bias because working Veterans will likely be unable to participate in the study. The added burden of a pretreatment assessment, MRI and tracking their activities in diaries also adds to the burden on the participants. This also will increase the potential for drop-out or partial treatment in the subjects which will threaten the ability to interpret the results with such a small sample size.

Inclusion of Women, Minorities and Children: Addressed in the proposal although no specific strategies are discussed to ensure that representative numbers of women and minorities are recruited. This could be a potential source of bias if women and minorities are not adequately represented in the sample.

Critique of Vertebrate Animals Section: Not applicable.

	Yes	No
Research with vertebrate animals?		X

Biohazards and Radioisotopes: Not applicable.

Budget: The budget is detailed and seems appropriate for the study.

Data Management and Access Plan (for data sharing, unscored)**:** Well described including monitoring security of data and procedures to maintain confidentiality.

Overall Strengths: This is a very relevant proposal for the funding mechanism because it is focused on validating a non-pharmacological intervention for chronic pain in Veterans and it has the potential to directly impact clinical care options available to providers within the VA. It is also very innovative by combining whole health interventions (yoga and a psychosocial intervention) with a novel method which appears to increase the efficacy of behavioral intervention (iTBS).

Overall Weaknesses: The study design is the major weakness because it does not allow the investigator the ability to understand if the iTBS has any added value above the Yoga/psychosocial intervention. This is important to establish given the costs of time and equipment in adding the iTBS as a standard of care. Although a manualized yoga intervention was selected, the reality is that it also is a combination of interventions (physical-yoga, psychosocial-cognitive). Although this intervention was selected because it was developed for use with TBI patients in rehabilitation settings, it may not be the ideal intervention for patients with MTBI who will not have significant functional impairments due to their MTBI. This intervention may not be challenging enough for MTBI patients if it was designed for patients with moderate to severe TBI. In addition, the number of weeks of treatment (6 weeks) seems very brief to have a significant impact on the multiple pain and functional measures included in the study. Consideration to the “dose” of treatment should be examined to see if this treatment is sufficient to

result in change in functioning (most physical therapists would not consider six sessions adequate for chronic pain). Examination of other yoga programs might be helpful.

CRITIQUE 3

Significance: Proposal seeks to develop a novel, combined neuromodulation and yoga neurorehabilitation intervention for Veterans with mTBI and chronic pain, and to examine the intervention's feasibility and acceptability. This program has the potential to be highly significant, as Veterans with mTBI have increased pain and compromise to quality of life. Additionally, the development of this program of rehabilitation has the potential to offer this vulnerable group of Veterans a treatment option for pain that does not involve opioids.

Approach: The Principal Investigator (PI) proposes a prospective, within-subject pilot study to develop a six week, six sessions combined iTBS+yoga treatment for TBI and comorbid pain. Twenty Veterans will be recruited who are over 22 years of age who have comorbid mTBI and chronic pain.

- There are concerns about the heterogeneity of the sample. Veterans over the age of 22 could encompass several war epochs and could result in groups that are wildly diverse with regard to age. Group exercise mixing generations of Veterans would not result in good group dynamics and could affect acceptability and feasibility. This reviewer would suggest a more targeted study population that is more homogenous and would not confound results with age.
- There are tests contained in the screening visit that do not address eligibility and are not outcomes (e.g., CVLT, NSI). What is the rationale for including these assessments? Table 2 indicates that they will be used to assess memory but that is not an outcome of this feasibility study. NSI and its purpose are not listed in Table 2. There are concerns that the NSI will be influenced by comorbidities in this Veteran sample.
- MRI will be conducted at the Center for Translational Imaging at Northwestern University. No offsite waiver to conduct these scans was included and the Director's letter specified that the work would be completed at the Hines VA Hospital. This may not be problem but without this information it is not possible to determine whether the critical MRI is feasible.
- The feasibility of the proposed treatment will be defined by enrollment and the number of sessions completed by each participant. Acceptability will be quantitatively assessed using satisfaction ratings (using a previously published tool relevant to this treatment/population) and with semi-structured clinical interviews with a subset of 10 Veterans. Why only 10? How will that subset of 10 be identified? The analysis of the qualitative interviews is complex, and there is concern whether the study team has any prior experience in this process.
- No concerns with the actual intervention.

General concerns: They do not have evidence that repeated iTBS will result in greater improvement over single session. Is there any plan to evaluate this hypothesis? Not demonstrated that iTBS (single or repeated) can be used safely with individuals with TBI (single case study reported to support this is insufficient). Alcohol use and alcohol use disorder is a common comorbidity in Veterans with mTBI and pain. The investigators may consider an assessment of alcohol to determine if use impact's their outcomes.

Innovation: The innovation of the study is in the novel combination of iTBS with yoga as a treatment for TBI and pain. Evidence suggests that the neural plasticity invoked by iTBS can last for up to 60 minutes following administration, which makes it ideal to precede a yoga session and potentially influence yoga's beneficial effects.

Investigator Qualifications: The investigative team is strong and has the expertise to carry out the proposed research.

Environment: The research environment appears excellent. Dr. Herrold and her team are well equipped with the necessary equipment/laboratory space.

Design/Scope: Design noted above. The timeline of the study is well delineated and seems feasible. Subject recruitment will proceed across a number of avenues that have been used in the past by these investigators, so there are no concerns.

Human Subjects: As mentioned, there is concern that the study population will be "Veterans over 22 years of age". This is potentially problematic, confounding age with feasibility/acceptability. The human subject section includes a paragraph about venipuncture (not relevant to this proposal). The biggest concern with iTBS is seizure induction. Will a physician be available in the event of an adverse effect?

Inclusion of Women, Minorities and Children: It is noted that both males and females will be included but it is not clear whether there will be equal representation.

Critique of Vertebrate Animals Section:

	Yes	No
Research with vertebrate animals?		X

Biohazards and Radioisotopes: Not applicable.

Budget: Appropriate.

Data Management and Access Plan (for data sharing, unscored)**:** Appropriate.

Overall Strengths: The major strengths of this proposal include the investigative team, resources and the proposed novel therapy combining iTBS and yoga. While the components of the study are not particularly innovative, combining them as a therapy to increase QOL in Veterans with mTBI and chronic pain is innovative.

Overall Weaknesses: The weaknesses have to do with the age of the proposed participants and the lack of detail in some of the study characteristics.

MEETING ROSTER

Rehabilitation Research and Development SPiRE Program
Rehabilitation Research and Development Parent IRG
Office of Research & Development
RRDS
04/23/2020

MEMBERS

AJIBOYE, ABIDEMI BOLU, PHD *
BIOMEDICAL ENGINEER
LOUIS STOKES CLEVELAND VA MEDICAL CENTER
ASSOCIATE PROFESSOR
FUNCTIONAL ELECTRICAL STIMULATION CENTER
CASE WESTERN RESERVE UNIVERSITY
CLEVELAND, OH 44106

ARYA, SHIPRA, MD *
CHIEF
DIVISION OF VASCULAR SURGERY
VA PALO ALTO HEALTH CARE SYSTEM
ASSOCIATE PROFESSOR OF SURGERY
STANFORD UNIVERSITY
STANFORD, CA 30322

BACHUS, KENT N., PHD *
RESEARCH BIOLOGIST
VA SALT LAKE CITY HEALTH CARE SYSTEM
RESEARCH PROFESSOR
DEPT OF ORTHOPAEDICS
UNIVERSITY OF UTAH
SALT LAKE CITY, UT 84108

BARR, GORDON ALFRED, PHD *
DIRECTOR AND BATTAGLIA ENDOWED CHAIR IN PEDIATRIC PAIN MEDICINE
DEPT OF ANESTHESIOLOGY & CRITICAL CARE MEDICINE
THE CHILDREN'S HOSPITAL OF PHILADELPHIA
& DEPT OF PSYCHOLOGY
UNIVERSITY OF PENNSYLVANIA
PHILADELPHIA, PA 19104

BETZ, KENDRA, MSPT *
CLINICAL PROGRAM SPECIALIST
NATIONAL CENTER FOR PATIENT SAFETY
OFFICE OF QUALITY, SAFETY AND VALUE
ROCKY MOUNTAIN REGIONAL VA MEDICAL CENTER
GLENDALE, CO 80246

BICKEL, C. SCOTT, PHD *
ASSOCIATE PROFESSOR
SCHOOL OF HEALTH
SAMFORD UNIVERSITY
BIRMINGHAM, AL 35233

BONDI, CORINA OANA, PHD *
ASSISTANT PROFESSOR
PHYSICAL MEDICINE
UNIVERSITY OF PITTSBURGH
PITTSBURGH, PA 15213

BOWDEN, MARK G., PHD *
PHYSICAL THERAPIST
RALPH JOHNSON VA MEDICAL CENTER
ASSOCIATE PROFESSOR
COLLEGE OF HEALTH PROFESSIONS
MEDICAL UNIVERSITY OF SOUTH CAROLINA
CHARLESTON, SC 29425

BOWEN, MARY E., PHD *
RESEARCH HEALTH SCIENCE SPECIALIST
CORPORAL MICHAEL J. CRESCENZ VA MEDICAL CENTER
ASSOCIATE PROFESSOR
SCHOOL OF NURSING
UNIVERSITY OF DELAWARE
NEWARK, DE 19716

BRENNER, LISA A, PHD *
PROFESSOR
DEPTS OF PHYSICAL MEDICINE &
REHABILITATION, NEUROLOGY & PSYCHIATRY
SCHOOL OF MEDICINE
UNIVERSITY OF COLORADO ANSCHUTZ MEDICAL CAMPUS
AURORA, CO 80045

BRITTON, PETER C, PHD *
PSYCHOLOGIST
CANANDAIGUA VA MEDICAL CENTER
CANANDAIGUA, NY 14424

CAMPANA, WENDY M., PHD *
RESEARCH HEALTH SCIENTIST
VA SAN DIEGO HEALHCARE SYSTEM
PROFESSOR
DEPARTMENT OF ANESTHESIOLOGY & NEUROSCIENCE
UNIVERSITY OF CALIFORNIA, SAN DIEGO
LA JOLLA, CA 92093

CARROLL, JOSEPH, PHD *
RICHARD O. SCHULTZ/RUTH WORKS PROFESSOR IN OPHTHALMOLOGY
THE MEDICAL COLLEGE OF WISCONSIN
MILWAUKEE, WI 53226

CHANG, JAMES, MD *
PROFESSOR AND CHIEF
VA PALO ALTO HEALTH CARE SYSTEM
DIVISION OF PLASTIC AND RECONSTRUCTIVE SURGERY
DEPTS OF SURGERY, ORTHOPEDIC, HAND &
MICROSURGERY
STANFORD UNIVERSITY MEDICAL CENTER
PALO ALTO, CA 94304

CHAPMAN, JULIE CATHERINE, PSYD *
DIRECTOR OF NEUROSCIENCE, NEUROLOGY SERVICE
WASHINGTON DC VA MEDICAL CENTER
ASSISTANT PROFESSOR OF NEUROLOGY
GEORGETOWN UNIVERSITY MEDICAL SCHOOL
WASHINGTON, DC 20422

CHOW, LESLEY W, PHD *
ASSISTANT PROFESSOR
DEPT OF BIOENGINEERING MATERIALS SCIENCE
& ENGINEERING
LEHIGH UNIVERSITY
BETHLEHEM, PA 18015

COMBS, MARTHA, PHD *
CLINICAL PSYCHOLOGIST
PRIMARY CARE-MENTAL HEALTH INTEGRATION
BALCONES HEIGHTS OUTPATIENT CLINIC
SOUTH TEXAS VETERANS HEALTH CARE SYSTEM
SAN ANTONIO, TX 78229

D'ANDREA, SUSAN ELIZABETH, PHD *
HEALTH SCIENCE SPECIALIST
PROVIDENCE VA MEDICAL CENTER
THE GAIT & MOTION ANALYSIS LABORATORY
THE CENTER FOR RESTORATIVE & REGENERATIVE
MEDICINE
PROVIDENCE, RI 02908

DAHIA, CHITRA L, PHD *
ASSISTANT PROFESSOR
DEPT OF CELL AND DEVELOPMENTAL BIOLOGY
WEILL CORNELL SCHOOL OF MEDICINE
NEW YORK, NY 10065

DAILEY, HANNAH LEE, PHD *
ASSISTANT PROFESSOR
DEPT OF ENGINEERING & MECHANICS
LEHIGH UNIVERSITY
BETHLEHEM, PA 18015

DANILOVICH, MARGARET K, PHD *
ASSISTANT PROFESSOR
PHYSICAL THERAPY AND HUMAN MOVEMENT SCIENCE
FEINBERG SCHOOL OF MEDICINE
NORTHWESTERN UNIVERSITY
EVANSTON, IL 60201

DAVEY, PINAKIN GUNVANT, PHD *
PROFESSOR & DIRECTOR OF RESEARCH
COLLEGE OF OPTOMETRY
WESTERN UNIVERSITY OF HEALTH SCIENCES
POMONA, CA 91766

DILLON, KIRSTEN HAWKINS, MS, PHD *
PSYCHOLOGIST
DURHAM VA MEDICAL CENTER
DURHAM, NC 27705

DRAKE, ANGELA, PHD *
CLINICAL PROFESSOR
UNIVERSITY OF CALIFORNIA - DAVIS
SAN DIEGO, CA 92122

ECKER, ANTHONY, PHD *
RESEARCH HEALTH SCIENTIST
MICHAEL E DEBAKEY VA MEDICAL CENTER
DEPT OF PSYCHIATRY AND BEHAVIORAL SCIENCES
BAYLOR COLLEGE OF MEDICINE
HOUSTON, TX 77030

FUDIM, MARAT, MD *
PROFESSOR
CLINICAL RESEARCH FELLOW, CARDIOLOGY
DUKE UNIVERSITY
DURHAM, NC 27713

FUGLEVAND, ANDREW J, PHD *
PROFESSOR
DEPARTMENT OF PHYSIOLOGY & NEUROSCIENCE
COLLEGE OF MEDICINE
UNIVERSITY OF ARIZONA
TUCSON, AZ 85721

GARDINIER, JOSEPH DANIEL, PHD *
ASSISTANT SCIENTIST
ORTHOPEDIC SURGERY
BONE & JOINT CENTER
HENRY FORD HEALTH SYSTEM
HENRY FORD HOSPITAL
DETROIT, MI 48202

GHILARDI, M. FELICE MARINA, MD *
PROFESSOR
DEPARTMENT OF PHYSIOLOGY, PHARMACOLOGY,
NEUROSCIENCE AND NEUROLOGY
CUNY MEDICAL SCHOOL
NEW YORK, NY 10031

GHOSH, MOUSUMI, PHD *
RESEARCH BIOLOGIST
MIAMI VA HEALTHCARE SYSTEM
RESEARCH ASSISTANT PROFESSOR
THE MIAMI PROJECT TO CURE PARALYSIS
UNIVERSITY OF MIAMI
MIAMI, FL 33136

GILLETT, JAMES, PHD *
ASSOCIATE PROFESSOR & DEPT CHAIR
DEPT OF HEALTH, AGING & SOCIETY
DEPT OF SOCIOLOGY
MCMASTER UNIVERSITY
HAMILTON, ON L8S 4M4
CANADA

GOLDISH, GARY DAVID, MD *
MEDICAL DIRECTOR
REHABILITATION ENGINEERING RESEARCH PROGRAM
MINNEAPOLIS VA HEALTH CARE SYSTEM
MINNEAPOLIS, MN 55417

GORGEY, ASHRAF, PHD *
PHYSICAL THERAPIST
SPINAL CORD INJURY & DISORDERS SERVICE
HUNTER HOLMES MCGUIRE VA MEDICAL CENTER
ASSISTANT PROFESSOR, DEPT OF PM&R
VIRGINIA COMMONWEALTH UNIVERSITY
RICHMOND, VA 23249

HAHN, JIN-OH, PHD *
ASSISTANT PROFESSOR
ROBERT E FISCHELL INSTITUTE FOR BIOMEDICAL DEVICES
APPLIED MATHEMATICS & STATISTICS
AND SCIENTIFIC COMPUTATION PROGRAM
UNIVERSITY OF MARYLAND
COLLEGE PARK, MD 20742

HART-HUGHES, STEPHANIE PT *
PRESIDENT AND CEO
HART FALL MITIGATION SOLUTIONS
TAMPA, FL 33602

HICKEY, DAVID, PHD *
ASSISTANT PROFESSOR
COLLEGE OF ENGINEERING
MICHIGAN STATE UNIVERSITY
EAST LANSING, MI 48824

HOFFMAN, AMY JUDE, PHD *
TENURED PROFESSOR
COLLEGE OF NURSING
UNIVERSITY OF NEBRASKA MEDICAL CENTER
OMAHA, NE 68198

HONAKER, JULIE ANN, PHD *
AUDIOLOGIST
HEAD AND NECK INSTITUTE
CLEVELAND CLINIC MAIN CAMPUS
CLEVELAND, OH 44195

HUGHES, ABBEY JEAN, PHD *
ASSISTANT PROFESSOR, ABPP-R
DEPT OF PHYSICAL MEDICINE & REHABILITATION
JOHNS HOPKINS UNIVERSITY SCHOOL OF MEDICINE
BALTIMORE, MD 21287

JENNINGS, JENNIFER, MD *
CLINICAL DIRECTOR WRIISC
DEPT OF NEUROSURGERY
VA PALO ALTO HEALTH CARE SYSTEM
PALO ALTO, CA 94304

JOHNSON, BENJAMIN, PHD *
ASSISTANT PROFESSOR
DEPT. OF ELECTRICAL AND COMPUTER ENGINEERING
BOISE STATE UNIVERSITY
BOISE, ID 83725

KEMP, STEPHEN WILLIAM PETER, PHD *
DIRECTOR
NEUROMUSCULAR LAB
ASSISTANT RESEARCH PROFESSOR
DEPTS OF SURGERY & BIOMEDICAL ENGINEERING
UNIVERSITY OF MICHIGAN
ANN ARBOR, MI 48109

KERBRAT, AMANDA, MSW *
RESEARCH SCIENTIST
DEPT OF PSYCHIATRY & BEHAVIORAL SCIENCES
UNIVERSITY OF WASHINGTON
SEATTLE, WA 98104

KERN, ROBERT S, PHD *
RESEARCH PSYCHOLOGIST
VA GREATER LA HEALTHCARE SYSTEM
DEPT OF PSYCHIATRY & BIOBEHAVIORAL SCIENCES
DAVID GEFFEN SCHOOL OF MEDICINE
UNIVERSITY OF CALIFORNIA, LOS ANGELES
LOS ANGELES, CA 90073

KLINE, ANTHONY E., PHD *
PROFESSOR
PHYSICAL MEDICINE & REHABILITATION
ASSOCIATE DIRECTOR OF REHABILITATION RESEARCH
UNIVERSITY OF PITTSBURGH SCHOOL OF MEDICINE
CHILDREN'S HOSPITAL OF PITTSBURGH
PITTSBURGH, PA 15224

KNIGHT, ELIZABETH, MD *
PHYSICIAN
ROCKY MOUNTAIN REGIONAL VA MEDICAL CENTER
AURORA, CO 80045

KOBAISSY, FIRAS H., PHD *
RESEARCH ASSISTANT PROFESSOR
DEPARTMENT OF PSYCHIATRY
UNIVERSITY OF FLORIDA COLLEGE OF MEDICINE
GAINESVILLE, FL 32610

KOFFEL, ERIN, PHD *
STAFF PSYCHOLOGIST
MINNEAPOLIS VA HEALTH CARE SYSTEM
ASSISTANT PROFESSOR
DEPARTMENT OF PSYCHIATRY
UNIVERSITY OF MINNESOTA
MINNEAPOLIS, MN 55417

KOONTZ, ALICIA M., PHD *
DIRECTOR FOR RESEARCH CAPACITY BUILDING
BIOMEDICAL ENGINEER
VA PITTSBURGH HEALTHCARE SYSTEM
PROFESSOR, DEPT. REHABILITATION SCIENCE & TECH.
UNIVERSITY OF PITTSBURGH
PITTSBURGH, PA 15206

LEE, JENNIFER ELLEN, PHD *
ADJUNCT ASSISTANT PROFESSOR
UNIVERSITY OF IOWA
IOWA CITY, IA 52242

LEMPKA, SCOTT F, PHD *
ASSISTANT PROFESSOR
DEPT OF BIOMEDICAL ENGINEERING & ANESTHESIOLOGY
UNIVERSITY OF MICHIGAN
ANN ARBOR, MI 48109

LEUNG, JACQUELINE M, MD *
PROFESSOR
DEPARTMENT OF ANESTHESIA &
PERIOPERATIVE CARE
UNIVERSITY OF CALIFORNIA SAN FRANCISCO
SAN FRANCISCO, CA 94143

LI, XUDONG J., PHD, MD *
ASSOCIATE PROFESSOR
DEPARTMENT OF ORTHOPEDIC SURGERY
& BIOMEDICAL ENGINEERING
SCHOOL OF MEDICINE
UNIVERSITY OF VIRGINIA
CHARLOTTESVILLE, VA 22908

LITTMAN, ALYSON, PHD *
RESEARCH HEALTH SCIENTIST
VA PUGET SOUND HEALTH CARE SYSTEM
RESEARCH ASSOCIATE PROFESSOR
SCHOOL OF PUBLIC HEALTH
UNIVERSITY OF WASHINGTON
SEATTLE, WA 98108

LIU, JIALING, PHD *
SURGEON
SAN FRANCSICO VA HEALTH CARE SYTEM
PROFESSOR
DEPARTMENT OF NEUROLOGICAL SURGERY
UNIVERSITY OF CALIFORNIA
SAN FRANCISCO, CA 94121

LUKKAHATAI, NADA, PHD *
ASSISTANT PROFESSOR
ACUTE AND CHRONIC CARE
JOHNS HOPKINS SCHOOL OF NURSING
BALTIMORE, MD 21205

MAHAN, MARK, MD *
ASSISTANT PROFESSOR
NEUROSURGERY
UNIVERSITY OF UTAH
SALT LAKE CITY, UT 84132

MANCINI, MARTINA, ENGD *
ASSISTANT PROFESSOR
DEPARTMENT OF NEUROLOGY
OREGON HEALTH & SCIENCE UNIVERSITY
PORTLAND, OR 97239

MARTINEZ, DIANA M, MD *
PROFESSOR
DEPT OF PSYCHIATRY
COLLEGE OF PHYSICIANS & SURGEONS
COLUMBIA UNIVERSITY
NEW YORK, NY 10032

MCCRAE, CHRISTINA S, PHD *
PROFESSOR
DEPARTMENT OF PSYCHIATRY & BEHAVIORAL SCIENCES
SCHOOL OF MEDICINE
UNIVERSITY OF MISSOURI
COLUMBIA, MO 65212

MCCREERY, RYAN W., PHD *
DIRECTOR OF RESEARCH
CENTER FOR AUDIOLOGY
BOYS TOWN NATIONAL RESEARCH HOSPITAL
OMAHA, NE 68131

MCDONALD, SCOTT D., PHD *
SCI NEUROPSYCHOLOGIST
HUNTER HOLMES MCGUIRE VA MEDICAL CENTER
ASSISTANT PROFESSOR
DEPTS OF PHYSICAL MED & REHAB & PSYCHOLOGY
VIRGINIA COMMONWEALTH UNIVERSITY
RICHMOND, VA 23249

MCGLINCHEY, REGINA, PHD *
DIRECTOR AND RESEARCH HEALTH SCIENTIST
VA BOSTON HEALTHCARE SYSTEM
ASSOCIATE PROFESSOR, DEPT OF PSYCHIATRY
HARVARD MEDICAL SCHOOL
WEST ROXBURY, MA 01886

MERCURI, JEREMY, PHD *
ASSOCIATE PROFESSOR
DEPT OF ORTHOPAEDIC ENGINEERING
& REGENERATIVE MEDICINE
CLEMSON UNIVERSITY
CLEMSON, SC 29634

MOHAN, SUBBURAMAN, PHD *
RESEARCH SCIENTIST
MUSCULOSKELETAL DISEASE CENTER
JERRY L. PETTIS MEMORIAL VA MEDICAL CENTER
LOMA LINDA, CA 92357

MOURAD, PIERRE D, PHD *
PROFESSOR
DIVISION OF ENGINEERING AND MATHEMATICS
DEPT OF NEUROLOGICAL SURGERY
DEPT OF BIOENGINEERING APPLIED PHYSICS
LABORATORY
UNIVERSITY OF WASHINGTON
SEATTLE, WA 98195

MUKHERJEE, PRATIK, PHD *
PROFESSOR & DIRECTOR
SAN FRANCISCO VA HEATLH CARE SYSTEM
DEPARTMENT OF RADIOLOGY & BIOENGINEERING
UNIVERSITY OF CALIFORNIA, SAN FRANCISCO
CENTER FOR IMAGING OF NEURODEGENERATIVE
DISEASES
SAN FRANCISCO, CA 94121

MURPHY, JOHN, MD *
CLINICAL LEAD
CONNECTED HEALTH CONNECTED CARE
HEALTH INFORMATICS
SOUTHEAST LOUISIANA VETERANS HEALTHCARE SYSTEM
NEW ORLEANS, LA 70119

NA, ANNALISA, PHD *
POSTDOCTURAL FELLOW
BIOMECHANICS AND MOVEMENT SCIENCES
UNIVERSITY OF TEXAS MEDICAL BRANCH
GALVESTON, TX 77555

NEITZ, JAY, PHD *
PROFESSOR
DEPT OF OPHTHALMOLOGY
UNIVERSITY OF WASHINGTON
SEATTLE, WA 98109

OLNEY, CHRISTINE M, PHD *
NURSE SCIENTIST
MINNEAPOLIS ADAPTIVE DESIGN & ENG
MINNEAPOLIS VA HEALTH CARE SYSTEM
MINNEAPOLIS, MN 55317

ONG, BINNAN, MD *
PHYSICIAN
SPINAL CORD INJURY SERVICE
LOUIS STOKES CLEVELAND VA MEDICAL CENTER
CLEVELAND, OH 44106

OTTOMANELLI, LISA PHD, PHD *
CLINICAL PSYCHOLOGIST
JAMES A. HALEY VETERANS' HOSPITAL
TAMPA, FL 33612

OWEISS, KARIM G, PHD *
PRE-EMINENT PROFESSOR
DEPARTMENT OF ELECTRICAL & COMPUTER ENGINEERING
HERBERT WESTHEIM COLLEGE OF ENGINEERING
THE MCKNIGHT BRAIN INSTITUTE
UNIVERSITY OF FLORIDA
GAINESVILLE, FL 32611

PEDERSEN, JESSICA PRESPERIN, MBA *
OT RESEARCH SCIENTIST
SHIRLEY RYAN ABILITY LAB
CHICAGO, IL 60611

PETRUSKA, JEFFREY C PHD, PHD *
ASSOCIATE PROFESSOR
DEPARTMENT OF ANATOMICAL SCIENCES &
NEUROBIOLOGY
UNIVERSITY OF LOUISVILLE
LOUISVILLE, KY 40202

PLESS KAISER, ANICA, PHD *
CLINICAL RESEARCH PSYCHOLOGIST
VA BOSTON HEALTHCARE SYSTEM
NATIONAL CENTER FOR PTSD
RESEARCH ASSISTANT PROFESSOR, PSYCHIATRY DEPT
BOSTON UNIVERSITY SCHOOL OF MEDICINE
BOSTON, MA 02130

PODLASEK, CAROL ANN, PHD *
ASSOCIATE PROFESSOR
DEPT OF UROLOGY, PHYSIOLOGY, & BIOENGINEERING
UNIVERSITY OF ILLINOIS AT CHICAGO
CHICAGO, IL 60612

POORE, SAMUEL, MD, PHD *
PHYSICIAN
WILLIAM S. MIDDLETON MEMORIAL VETERANS HOSPITAL
PROFESSOR
DIVISION OF PLASTIC SURGERY
UNIVERSITY OF WISCONSIN
MADISON, WI 53792

PUNDIK, SVETLANA, MD *
PHYSICIAN
LOUIS STOKES CLEVELAND VA MEDICAL CENTER
ASSOCIATE PROFESSOR
DEPT OF NEUROLOGY SCHOOL OF MEDICINE
CASE WESTERN RESERVE UNIVERSITY
CLEVELAND, OH 44106

RAMDHANI, RITESH, MD *
ASSISTANT PROFESSOR
DEPT OF NEUROLOGY
NEW YORK UNIVERSITY LANGONE HEALTH
NEW YORK, NY 11040

RAY, ANDREW DONALD, PHD *
ASSOCIATE PROFESSOR
DEPARTMENT OF CANCER PREVENTION & CONTROL
ROSWELL PARK COMPREHENSIVE CANCER CENTER
BUFFALO, NY 14263

REILLY, ERIN, PHD *
CLINICAL RESEARCH PSYCHOLOGIST
EDITH NOURSE ROGERS MEMORIAL VETERANS HOSPITAL
ASSISTANT PROFESSOR OF PSYCHIATRY
UNIVERSITY OF MASSACHUSETTS MEDICAL SCHOOL
ARLINGTON, MA 02474

RENGA, VIJAY, MD *
NEUROLOGIST
DARTMOUTH-HITCHCOCK MEDICAL CENTER
LEANON, NH 03756

RIDGEL, ANGELA L, PHD *
TENURED ASSOCIATE PROFESSOR
SCHOOL OF HEALTH SCIENCE
EXERCISE PHYSIOLOGY
KENT STATE UNIVERSITY
KENT, OH 44242

ROBINSON, WILLIAM H, PHD, MD *
PHYSICIAN
GERIATRIC RESEARCH, EDUCATION & CLINICAL CENTER
VA PALO ALTO HEALTH CARE SYSTEM
PROFESSOR, DIVISION OF IMMUNOLOOGY &
RHEUMATOLOGY
STANFORD UNIVERSITY
PALO ALTO, CA 94304

RUGGIERI, MICHAEL RAYMOND SR, PHD *
PROFESOR
DEPARTMENT OF ANATOMY & CELL BIOLOGY
LEWIS KATZ SCHOOL OF MEDICINE
TEMPLE UNIVERSITY
PHILADELPHIA, PA 19140

RYAN, ALICE S., PHD *
PROFESSOR
VA MARYLAND HEALTH CARE SYSTEM
SCHOOL OF MEDICINE
DIVISION OF GERIATRICS & GERIATRIC MEDICINE
UNIVERSITY OF MARYLAND
BALTIMORE, MD 21201

SAJATOVIC, MARTHA X, MD *
PROFESSOR OF PSYCHIATRY & NEUROLOGY AND WILLARD
BROWN CHAIR IN NEUROLOGICAL OUTCOMES
DEPT OF PSYCHIATRY & NEUROLOGY
SCHOOL OF MEDICINE
CASE WESTERN RESERVE UNIVERSITY
CLEVELAND, OH 44106

SALISBURY, JOSEPH PETER, PHD *
HEAD OF DIGITAL THERAPEUTICS COGNIVIVE, INC.
QUINCY, MA 02171

SCHNELLMANN, RICK G, PHD *
HEALTH SCIENCE SPECIALIST
SOUTHERN ARIZONA VA HEALTHCARE SYSTEM
PROFESSOR & DEAN
DEPARTMENT OF PHARMACOLOGY & TOXICOLOGY
UNIVERSITY OF ARIZONA
TUCSON, AZ 85721

SCHROEDER, KAREN ELIZABETH, PHD *
POSTDOCTORAL RESEARCH SCIENTIST
GREENE SCIENCE CENTER
COLUMBIA UNIVERSITY
NEW YORK, NY 10027

SEEL, RON, PHD *
EXECUTIVE DIRECTOR
CENTER FOR REHABILITATION SCIENCE & ENGINEERING
PROFESSOR
DEPT OF PHYSICAL MEDICINE & REHABILITATION
VIRGINIA COMMONWEALTH UNIV, SCHOOL OF MEDICINE
RICHMOND, VA 23298

SEGIL, JACOB, PHD *
VA EASTERN COLORADO HEALTHCARE SYSTEM
INSTRUCTOR
ENGINEERING PLUS
UNIVERITY OF COLORADO BOULDER
BOULDER, CO 80309

SHARMA, BLANKA, PHD *
ASSISTANT PROFESSOR
DEPARTMENT OF BIOMEDICAL ENGINEERING
UNIVERSITY OF FLORIDA
GAINESVILLE, FL 32611

SHARMA, NITIN, PHD *
ASSOCIATE PROFESSOR
DEPT. OF MECHANICAL ENGINEERING & MATERIAL
SCIENCE
UNIVERSITY OF PITTSBURGH
PITTSBURGH, PA 15213

SIKDAR, SIDDHARTHA, PHD *
ASSOCIATE PROFESSOR
DEPTS OF BIOENGINEERING &
ELECTRICAL & COMPUTER ENGINEERING
GEORGE MASON UNIVERSITY
FAIRFAX, VA 22030

SILDER, AMY, PHD *
RESEARCH ENGINEER
WARFIGHTER PERFORMANCE DIVISION
NAVAL MEDICAL CENTER SAN DIEGO
SAN DIEGO, CA 92134

SOLINSKY, RYAN, MD *
PHYSICIAN-SCIENTIST
SPINAL CORD INJURY MEDICINE
SPAULDING REHABILITATION HOSPITAL
CHARLESTOWN, MA 02129

STANTON, AMELIA M., PHD *
CLINICAL RESEARCH FELLOW
MASSACHUSETTS GENERAL HOSPITAL
HARVARD MEDICAL SCHOOL
BOSTON, MA 02114

STEIN, DONALD G., PHD *
ASA G. CANDLER PROFESSOR
DEPT OF EMERGENCY MEDICINE
BRAIN RESEARCH LABORATORY
EMORY UNIVERSITY SCHOOL OF MEDICINE
ATLANTA, GA 30322

SUBRAMANIAM, BALACHUNDHAR, MD *
ASSOCIATE PROFESSOR
DEPT OF ANAESTHESIA
HARVARD MEDICAL SCHOOL
BOSTON, MA 02215

SUN, VIRGINIA CHIH-YI, PHD *
ASSOCIATE PROFESSOR
DIVISION OF NURSING RESEARCH AND EDUCATION
DEPARTMENT OF POPULATION SCIENCES
CANCER CONTROL AND POPULATION SCIENCES PROGRAM
CITY OF HOPE
DUARTE, CA 91010

TAKAHASHI, KOTA, PHD *
ASSISTANT PROFESSOR
DEPARTMENT OF BIOMECHANICS
UNIVERSITY OF NEBRASKA AT OMAHA
OMAHA, NE 68182

TAN, ANDREW MICHAEL, PHD *
ASSOCIATE RESEARCH SCIENTIST
VA CONNECTICUT HEALTHCARE SYSTEM
DEPARTMENT OF NEUROLOGY
SCHOOL OF MEDICINE
YALE UNIVERSITY
WEST HAVEN, CT 06516

TAPPEN, RUTH M, RN *
CHRISTINE E. LYNN EMINENT SCHOLAR AND PROFESSOR
COLLEGE OF NURSING
FLORIDA ATLANTIC UNIVERSITY
BOCA RATON, FL 33431

TEITZ, TAL, PHD *
ASSISTANT PROFESSOR
DEPT OF PHARMACOLOGY
SCHOOL OF MEDICINE
CREIGHTON UNIVERSITY
OMAHA, NE 68178

TROY, KAREN L, PHD *
ASSOCIATE PROFESSOR
BIOMEDICAL ENGINEERING
WORCESTER POLYTECHNIC INSTITUTE
WORCESTER, MA 01609

TRUMBOWER, RANDY, MS, PHD *
DIRECTOR
INSPIRE LAB FOR SENSORIMOTOR REHABILITATION ENGIN
HARVARD MEDICAL SCHOOL
SPAULDING REHABILITATION HOSPITAL
CAMBRIDGE, MA 02138

TSAO, JACK W, PHD, MD *
STAFF PHYSICIAN
DEPARTMENT OF NEUROLOGY
MEMPHIS VA MEDICAL CENTER
PROFESSOR
UNIVERSITY OF TENNESSEE HEALTH SCIENCE CENTER
MEMPHIS, TN 38163

TUNIK, EUGENE, PHD *
PROFESSOR
DEPARTMENT OF PHYSICAL THERAPY, MOVEMENT &
REHABILITATION SCIENCE
BOUVE COLLEGE OF HEALTH SCIENCES
NORTHEASTERN UNIVERSITY
BOSTON, MA 02115

TWAMLEY, ELIZABETH W, PHD *
STAFF NEUROPSYCHOLOGIST
VA SAN DIEGO HEALTHCARE SYSTEM
SAN DIEGO, CA 92103

TYLER, DUSTIN J., PHD *
RESEARCH CAREER SCIENTIST
ASSOC DIRECTOR, ADVANCED PLATFORM TECHNOLOGY
CTR
LOUIS STOKES CLEVELAND VA MEDICAL CENTER
DEPT OF BIOMEDICAL ENGINEERING
CASE WESTERN RESERVE UNIVERSITY
CLEVELAND, OH 44106

VAN 'T WOUT-FRANK, MASCHA, PHD *
AFFILIATE INVESTIGATOR
PROVIDENCE VA MEDICAL CENTER
ASSISTANT PROFESSOR
DEPT OF PYSCHIATRY & HUMAN BEHAVIOR
BROWN UNIVERSITY
PROVIDENCE, RI 02906

VASUNILASHORN, SARINNAPHA, PHD *
INSTRUCTOR IN MEDICINE
DIVISION OF GENERAL MEDICINE & PRIMARY CARE
BETH ISRAEL DEACONESS MEDICAL CENTER
HARVARD MEDICAL SCHOOL
BOSTON, MA 02215

VITIELLO, MICHAEL V., PHD *
PROFESSOR
DEPARTMENT OF PSYCHIATRY &
BEHAVIORAL SCIENCES
UNIVERSITY OF WASHINGTON
SEATTLE, WA 98195

WANG, JAMES H-C., PHD *
PROFESSOR & DIRECTOR
DEPTS OF ORTHOPAEDIC SURGERY
& BIOENGINEERING & MECHANICAL ENGINEERING
UNIVERSITY OF PITTSBURGH
PITTSBURGH, PA 15213

WEAVER, ASHLEY, PHD *
ASSISTANT PROFESSOR
DEPARTMENT OF BIOMEDICAL ENGINEERING
WAKE FOREST UNIVERSITY SCHOOL OF MEDICINE
WINSTON-SALEM, NC 27157

WEIR, RICHARD FERGUS FFRENCH, PHD *
RESEARCH ASSOCIATE PROFESSOR
DEPT OF BIOENGINEERING
VA EASTERN COLORADO HEALTH CARE SYSTEM
DIRECTOR, BIOMECHATRONICS DEVELOPMENT
LABORATORY
UNIVERSITY OF COLORADO, DENVER
AURORA, CO 80045

WEITZEL, WILLIAM F, MD *
ASSOC. CHIEF OF STAFF, RESEARCH
VA ANN ARBOR HEALTHCARE SYSTEM
PROFESSOR OF NEPHROLOGY
UNIVERSITY OF MICHIGAN MEDICAL CENTER
ANN ARBOR, MI 48105

WITEK, LUKASZ, PHD *
ASSISTANT PROFESSOR
DIRECTOR OF CRANIOMAXILLOFACIAL ORTHOPAEDIC
AND REGENERATION APPLICATIONS LAB
NEW YORK UNIVERSITY COLLEGE OF DENTISTRY
NEW YORK, NY 10010

WOLF, JOHN ALLEN, PHD *
RESEARCH ASSIST PROFESSOR OF NEUROSURGERY
CORPORAL MICHAEL J. CRESCENZ VA MEDICAL CTR
ASSISTANT PROFESSOR
UNIVERSITY OF PENNSYLVANIA
PHILADELPHIA, PA 19104

XU, QINGGUO, PHD *
ASSISTANT PROFESSOR
DEPT OF PHARMACEUTICS
& CENTER FOR PHARMACEUTICAL ENGINEERING &
SCIENCES
SCHOOL OF PHARMACY
VIRGINIA COMMONWEALTH UNIVERSITY
RICHMOND, VA 23284

ZENI, JOSEPH ADAM JR, PHD *
ASSOCIATE PROFESSOR
DEPARTMENT OF REHABILITATION & MOVEMENT SCIENCES
SCHOOL OF HEALTH PROFESSIONS
RUTGERS UNIVERSITY
NEWARK, NJ 07107

SCIENTIFIC REVIEW OFFICER

BENTON-GROVER, KRISTY
MANAGEMENT AND PROGRAM ANALYST
DEPARTMENT OF VETERANS AFFAIRS
VETERANS HEALTH ADMINISTRATION
OFFICE OF RESEARCH AND DEVELOPMENT
REHABILITATION RESEARCH & DEVELOPMENT SERVICE
WASHINGTON, DC 20420

* Temporary Member. For grant applications, temporary members may participate in the entire meeting or may review only selected applications as needed.

Consultants are required to absent themselves from the room during the review of any application if their presence would constitute or appear to constitute a conflict of interest.
